# Aucubin inhibited lipid accumulation and oxidative stress via Nrf2/HO‐1 and AMPK signalling pathways

**DOI:** 10.1111/jcmm.14293

**Published:** 2019-04-04

**Authors:** Bingyu Shen, Chenxu Zhao, Yue Wang, Yi Peng, Jiaqi Cheng, Zheng Li, Lin Wu, Meiyu Jin, Haihua Feng

**Affiliations:** ^1^ Key Laboratory of Zoonosis Ministry of Education College of Veterinary Medicine Jilin University Changchun Jilin PR China; ^2^ Department of Pediatric Hematology The First Hospital of Jilin University Changchun Jilin PR China; ^3^ Department of Hematology The First Hospital of Jilin University Changchun Jilin PR China

**Keywords:** Aucubin, NAFLD, Nrf2, tyloxapol

## Abstract

Aucubin (AU) is the main active ingredient of *Aucuba japonica* which has showed many positive effects such as anti‐inflammation and liver protection. Non‐alcoholic fatty liver disease (NAFLD) is the most common cause of chronic liver disease. In this research, we explored the effects of AU on the tyloxapol‐induced NAFLD in mice and apolipoprotein C‐III (apoC‐III) induced‐3T3L1 cells. Tyloxapol (300 mg/kg) was injected to C57BL/6 mice with aucubin. The differentiated 3T3‐L1 cells were treated with or without aucubin after stimulation of apoC‐III (100 μg/mL). In results, aucubin inhibited hyperlipidaemia, oxidative stress and inflammation by influencing the content of total cholesterol (TC), triglyceride (TG), low density lipoprotein (LDL), very low density lipoprotein (VLDL), myeloperoxidase (MPO), superoxide dismutase (SOD), tumour necrosis factor receptor‐α (TNF‐α), interleukin‐1β (IL‐1β), and IL‐6 in blood. AU activated NF‐E2‐related factor 2 (Nrf2), peroxisome proliferator‐activated receptor α (PPARα), PPARγ and hemeoxygenase‐1 (HO‐1) and promoted the phosphorylation of adenosine 5′‐monophosphate‐activated protein kinase (AMPKα), AMPKβ, acetyl‐CoA carboxylase (ACC) and protein kinase B (AKT). In conclusion, AU performed the function of hypolipidaemic by its obvious anti‐inflammation and antioxidant activity, which may become a kind of new drug targeting at NAFLD.

## 1 INTRODUCTION

Non‐alcoholic fatty liver disease (NAFLD) is an increasingly recognized cause of liver‐related diseases and death. It represents a series of liver diseases characterized by macrovesicular steatosis in the case of alcohol intake (less than 40 g of ethanol per week) that is generally considered to be harmful to the liver. Although the relationship between hepatic alveolar steatosis and inflammatory changes and fibrosis in obese patients has been known for decades, it is a fact that has been ignored clinically.^1^ NAFLD increases risk of type 2 diabetes mellitus (T2DM), cardiovascular (CVD) and cardiac diseases, and chronic kidney disease (CKD).^2^ NAFLD can develop into non‐alcoholic steatohepatitis (NASH) and eventually progress into hepatocellular carcinoma (HCC).^3^ It could activate some related inflammatory or stress‐response molecules such as nuclear factor‐kappa B (NF‐κB), phosphatase and tensin homolog (PTEN) when HCC occurs.^4^ It also has been reported that toll‐like receptor 4 (TLR‐4), which is an activator of NF‐κB, may up‐regulate expression by gut microbiota‐derived ligand (like lipopolysaccharide).^5^ In the progress of NAFLD, it is often accompanied by ectopic fat accumulation and this is usually associated with increasing secretion of hepatokines,^6^, ^7^ gluconeogenesis, decreased glycogen synthesis and inhibition of insulin signalling.^8^

Transcription factor nuclear factor erythrocyte 2‐related factor 2 (Nrf2) is a positive regulator of a group of gene expression involved in antioxidant/electrophilic stress protection. Nrf2 regulates downstream antioxidant stress genes such as hemeoxygenase‐1 (HO‐1) and superoxide dismutase (SOD). It is a key regulator of cellular stress defense mechanisms by binding to antioxidant redox elements (ARE) when challenged by cellular stress.^9^, ^10^ In rodents, Nrf2 is also involved in hepatic fatty acid metabolism as a negative regulator of genes that promote hepatic ossification. Several recent works suggest the nuclear receptors peroxisome proliferator‐activated receptor (PPAR) α and PPARγ are involved in anti‐inflammation reactions in atheroma associated cells.^11^, ^12^, ^13^, ^14^, ^15^, ^16^ PPAR regulates gene expression by acting as a transcription factor for specific ligands. PPARα and PPARγ catalysts could reduce inflammatory proteins such as adhesion molecules, cytokines, chemokines, in monocytes/macrophages, ECs and vascular smooth muscle cells.^17^ In addition, it has been revealed that PPAR activators can limit experimental atherosclerosis in animal models, although its expression is considered to be restricted to the tissues like liver and fat.^18^, ^19^

AMP‐activated protein kinase (AMPK) is a key regulator of cellular energy homeostasis. It can sense cellular ATP starvation and is an important regulator of autophagy.^20^ AMPK is prevalent as a heterotrimeric complex containing α catalytic subunits and β and γ regulatory subunits, which occur in multiple subtypes (α1/α2; β1/β2; γ1/γ2/γ3) encoded by different genes.^21^ In skeletal muscle and liver, AMPK activates fatty acid oxidation by inhibiting the acetyl‐CoA carboxylase (ACC1)/ACC2 subtype of ACC, thereby reducing malonyl‐CoA, a substance that inhibits fatty acid entry into mitochondria.^22^, ^23^ AMPK promotes the expression of the tricarboxylic acid cycle enzyme, as well as the mitochondrial biological origin, which achieves transcriptional co‐activator peroxisome proliferator‐activated receptor gamma coactivator 1‐α (PGC‐1α) by increasing expression/activity.^24^ PGC‐1α could coactive several transcription factors associated with oxidative stress, including Nrf2 and PPARα.^21^

**Figure 1:**
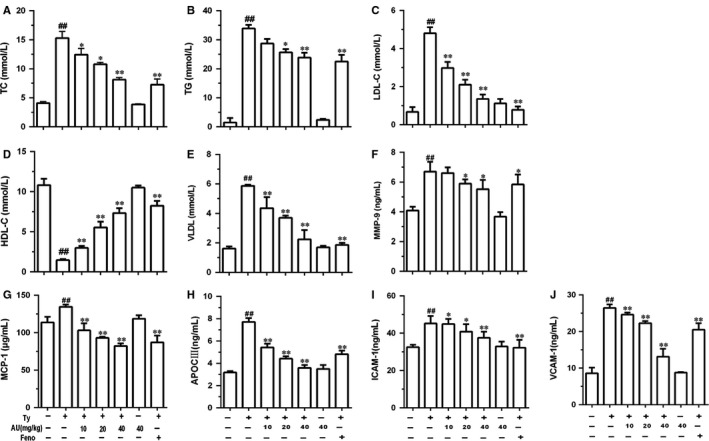
Aucubin decreased the accumulation of total cholesterol (TC), triglyceride (TG), low density lipoprotein (LDL‐C), very low density lipoprotein (VLDL), MMP‐9, MCP‐1, apoC‐III, ICAM‐1 and VCAM‐1 induced by tyloxapol in mice. C57BL/6 mice were received aucubin (10, 20, 40 mg/kg) prior to 1 h tyloxapol (300 mg/kg) injection and collecting serum was detected. A, The levels of TC. B, The levels of TG. C, The levels of LDL‐C. D, The levels of HDL‐C. E, The levels of VLDL. F, The levels of MMP‐9. G, The levels of MCP‐1. H, The levels of apoC‐III. I, The levels of ICAM‐1. J, The levels of VCAM‐1. The values represent mean ± SEM of three independent experiments and differences between mean values were assessed by Student's *t* test. ^##^ *P* < 0.01 versus the control group, *P < 0.05 and **P < 0.01 versus the Ty‐treated group

Aucubin (1, 4, 5, 7 a‐tetra‐5‐hydroxy‐7—(hydroxymethyl) cyclopenta (c) pyran 1‐yl‐β‐D‐glucopyranoside, Figure 1), a natural compound extracted from various plants including *Aucuba indica* and *Eucommia* leaves, is proven to have a variety of pharmacological effects, especially anti‐inflammatory effects.^25^, ^26^, ^27^, ^28^, ^29^, ^30^, ^31^ Aucubin could inhibit the RNA and protein synthesis in the liver of mice, and protect liver from the damage caused by carbon tetrachloride or alpha‐amanitin in mice and rats, and has antibacterial activity.^25^, ^26^ In this experiment, we examined the potential regulatory effects of aucubin on NAFLD via Nrf2 and AMPK family. It was found that aucubin significantly regulated the lipid accumulation and oxidative stress both in vivo and in vitro to inhibit the damage caused by NAFLD.

## 2 MATERIALS AND METHODS

### 2.1 Reagents and chemical

Nrf2 (Cat.66504), Lamin B (Cat.66095) and β‐actin (Cat.60008) antibodies were purchased from Proteintech (Boston, MA, USA). PPARα (ab8934) and PPARγ (ab191407) antibodies were provided by Abcam (Cambridge, MA, USA). Antibodies against HO‐1 (D60G11) (5853S), AMPKα (5831T), p‐AMPKα (2535T), AMPKβ (4150T), p‐AMPKβ (4186T), adenosine ACC (3676T), p‐ACC (11818T) were purchased from Cell Signal Technology (Boston, MA, USA). Protein kinase B (AKT) (AF6261), p‐AKT (Thr 308) (AF3262), GAPDH (AF7021) were purchased from Affinity (OH, USA). HRP‐conjugated goat anti‐rabbit and goat anti‐mouse antibodies were provided by Boster (California, USA). FG super sensitive ECL luminescence reagent was provided by Meilunbio (Dalian, China).

### 2.2 Animals

Male C57/BL6 mice (6‐8 weeks), weighing approximately 20 g, were purchased from Liaoning Changsheng Biotechnology (Liaoning, China). The mice were fed a standard diet and housed under a specific pathogen free (SPF) condition (temperature: 24 ± 1°C, relative humidity: 40%–80%, n = 3 per cage). The animal studies are reported as recommended by the ARRIVE guidelines.^32^ All animal experiments were performed in accordance with the National Institutes of Health (NIH) guide for the Care and Use of Laboratory Animals and approved by the Jilin University animal administration committee.

### 2.3 In vivo study

#### 2.3.1 Aucubin treatment of mice

Aucubin (HPLC ≥ 98%) was purchased from Meilun Bio‐technology (Dalian, Liaoning, China). After 3 days of feeding, C57BL/6 mice were randomly divided into seven groups (n = 21, 3 for each group): Control group, tyloxapol group, tyloxapol + aucubin 10 mg/kg group, tyloxapol + aucubin 20 mg/kg group, tyloxapol + aucubin 40 mg/kg group, tyloxapol + fenofibrate (100 mg/kg) group and aucubin (40 mg/kg) group. The mice were fasted for 24 h prior to the administration of the drug. The mice in tyloxapol + aucubin groups (10, 20, 40 mg/kg) and aucubin group (40 mg/kg) were given intraperitoneal injection of aucubin in the second day. After 1 hour, mice in tyloxapol group and tyloxapol + aucubin (10, 20, 40 mg/kg) groups were given tyloxapol (500 mg/kg). The mice were killed painlessly and then the blood and liver of all groups were collected for the following experiments after 24 hours. Each experiment was repeated three times.

#### 2.3.2 Measurement of blood indexes in serum

All groups of mice were taking serum and the levels of TG, TC, LDL‐C, HDL‐C (high density lipoprotein), VLDL (Jiancheng Nanjing, Jiangsu, China), MMP‐9 (matrix metalloprotein), MCP‐1 (monocyte chemotactic protein) (R&D Systems, MN, USA), apoC‐III (Sigma, MO, USA), ICAM‐1 (intercellular adhesion molecule) and VCAM‐1 (vascular cell adhesion protein) (Mlbio, Shanghai, China) were detected in accordance with the kit manual operation.

#### 2.3.3 Measurement of SOD in serum

The serum was collected after 24 hours of aucubin treatment. The serum of all groups was pre‐tested to determine the optimal inhibition rate. The preliminary results showed that the inhibition rate was 46% when the serum was diluted 15 times for the following experiments. The SOD activity was calculated following the instruction of kit (Jiancheng Nanjing, Jiangsu, China).

#### 2.3.4 Measurement of MPO in liver

Fifty milligrams liver of each mouse was accurately weighed after 24 hours of aucubin treatment. The activity of MPO was calculated according to the kit's instruction (Jiancheng Nanjing, Jiangsu, China).

#### 2.3.5 Measurement of TNF‐α, IL‐1β and IL‐6 in serum

The levels of TNF‐α, IL‐1β and IL‐6 in serum were detected by ELISA kits according to the manufacturer's instruction (BioLegend, CA, USA).

#### 2.3.6 Western blotting

The liver (about 20 mg) was ground using Electric Tissue Grander (Tiangen OSE‐Y30). Then RIPA buffer and PMSF (1 mM) were added into the liver homogenate separated for 30 minutes and centrifuged with 13 000 g at 4°C for 10 minutes. Protein concentrations were determined by Pierce BCA protein assay kit (Thermo, USA). Twenty micrograms of each protein sample was transferred onto a polyvinylidene difluoride (PVDF) membrane after separation from 10% SDS‐polyacrylamide‐gel. Then, all membranes were blocked in 5% skim milk with TBST and shaken for 1 hour at room temperature. The membranes were washed four times, once 5 minutes. The primary antibodies (1:1000) were incubated with membranes at 4°C overnight. Membranes were washed as mentioned above and soaked into the secondary antibodies (1:5000), shaking at room temperature for 45 minutes and washed. Finally, the membranes were visualized by chemiluminescence (ECL) Western blotting detection system (Tanon, China) and analysed by Image Pro Plus software.

#### 2.3.7 Oil‐red staining for liver

The middle of the fresh liver lobe was put into OCT embedding and storing at −20°C. The tissue sections were made by freezing microtome using embedded wax. The sections were placed in 75% ethanol for 3 minutes and 0.3% oil red dye for 10 minutes. The sections were placed in the essence of cypress for 15 seconds and covered by the glass slides. The sections were observed under the microscope.

#### 2.3.8 Haematoxylin and eosin staining for liver

The left lobe of liver was collected after the mice were killed and put into 4% formaldehyde buffer. The tissues were embedded in paraffin, sliced and stained with haematoxylin and eosin. The slices were observed with a light microscope.

#### 2.3.9 Measurement of liver index

The weight and total liver of each mouse were measured accurately. Liver index = liver weight/body weight, and the mean value was calculated.

#### 2.3.10 Observation of blood character

After 24 hours of the treatment, the blood was taken and put at 4°C in 1.5 mL EP overnight. The images were collected.

### 2.4 In vivo study

#### 2.4.1 Cell culture

3T3‐L1 cells (American Type Culture Collection‐ATCC, ATCC^®^ CRL‐3242). were cultured in 1% glutamine DMEM containing 10% FBS, 100 U and 100 U/mL penicillin streptomycin and 2.5 mmol/L HEPES (Invitrogen‐Gibco, NY, USA), placed in 5% CO_2_ incubated in the constant temperature of 37°C incubator.

#### 2.4.2 3T3‐L1 cell differentiation

3T3‐L1 cells were inoculated with complete culture medium in 35 mm petri dishes (1 × 10^6^/well) at 37 °C and 5% CO_2_ incubator. When the cells grew to 80%–90%, the medium was replaced with it containing 0.5 mM IBMX (storage density of 0.5 mol/L), 1 μmol/L DEX (storage density of 2.5 mol/L), and 1.7 μmol/L insulin (storage density of 1.7 mol/L). The medium was exchanged with the medium containing 1 μmol/L insulin after 48 hours and cultured for another 48 hours. According to the state of cell growth and differentiation, complete culture medium should be replaced every 48 hours to continue culture. After 8‐10 days, the complete differential cells could be used for subsequent experiments.

#### 2.4.3 Cell count kits ‐8

The differential 3T3‐L1 cells were cultured in the 96‐well plate (4 × 10^5^ cells/mL) for 1 hour. DMEM medium was added to the control group, and 20, 35, 70, 140 and 280 μg/mL aucubin were added to the aucubin group, respectively. Each group was added with 10 μL CCK‐8 and cultured for 3‐4 hours. OD value was determined by 450 nm enzyme assays.

#### 2.4.4 Measurement of TG, TNF‐α, IL‐1β and IL‐6

The differential 3T3‐L1 cells were incubated into 6‐well plate (2 × 10^4^ cells/well). Aucubin (35, 70, 140 μg/mL) was added to the different concentrations of drug groups (35, 70, 140 μg/mL). After 1 hour, apoC‐III (100 μg/mL) was added to all groups except the control group. The equal volume medium was added to the control group. After 18 hours, the cell supernatant was absorbed and centrifuged to detect the levels of TNF‐α, IL‐1β and IL‐6. The next steps followed the manufacturer's instructions (BioLegend, CA, USA). The lysis cells were used for testing content of TG according to the manufacturer's instructions (Applygen, Beijing, China).

#### 2.4.5 Detection of the expression of mRNA

Software Primer Express 5.0 was used to design the sequences of TNF‐α, IL‐1β, IL‐6 and β‐actin (Table 1). Trizol was used to extract the total RNA from the differential 3T3‐L1 cells treated with aucubin and apoC‐III. Total RNA content and purity were measured by K5500 spectrophotometer. About 5 μg total RNA is used for reverse transcription to cDNA using Prime Script RT reagent kit (TaKaRa Biotechnology Co., Ltd.). The mRNA expression levels were evaluated by qRT‐PCR using the SYBR Green QuantiTect RT‐PCR Kit (TaKaRa Biotechnology Co., Ltd.) and the 7000 Fast Real‐Time PCR System (Applied Biosystems). The relative expression of each gene was normalized to β‐actin.

**Table 1:** Oligonucleotide Primers Used for qPCR

Gene	Primer sequences (5′‐3′)	Length (bp)	Sequence number
TNF‐α	For CCTATGTCTCAGCCTCTTCTCAT Rev CACTTGGTGGTTTGCTACGA	214	NM_013693.3
IL‐1β	For ACCTGTGTCTTTCCCGTGG Rev TCATCTCGGAGCCTGTAGTG	162	XM_006498795.3
IL‐6	For GGAGAGGAGACTTCACAGAGGA Rev ATTTCCACGATTTCCCAGAGA	103	NM_031168.2
β‐actin	For GGCTGTATTCCCCTCCATCG Rev CCAGTTGGTAACAATGCCATGT	154	NM_007393.5

#### 2.4.6 Western blot

The differential 3T3‐L1 cells were used to extract protein. The method was the same as mentioned in *Western blotting*.

#### 2.4.7 Statistics analysis

The obtained data were analysed using IBM SPSS Statistics 19.0 and the results were shown as mean plus or minus standard deviation. ONE way‐ANOVA was used for multi‐group comparisons and Student's *t* test was used between the two groups. ^##^ *P* < 0.01 indicates a significant difference from the control group. *P < 0.05 indicates a significant difference from the model group and **P < 0.01 indicates a significant difference from the model group.

## 3 RESULTS

### 3.1 The effects of aucubin in mice

#### 3.1.1 Aucubin decreased the accumulation of TC, TG, LDL‐C and VLDL induced by tyloxapol

The results showed that TC, TG, LDL‐C and VLDL content of mice in tyloxapol group increased significantly compared with the control group (*P* < 0.01), and the content of HDL‐C decreased significantly (*P* < 0.01). The content of TC, TG, LDL‐C and VLDL in aucubin groups (10, 20 and 40 mg/kg) decreased significantly compared with the tyloxapol group (*P* < 0.05 or *P* < 0.01), while the content of HDL‐C increased significantly (*P* < 0.01). After the treatment with fenofibrate, all four indicators showed a significant decrease (Figure 1A‐C and E; *P* < 0.01) and a significant increase in HDL‐C (Figure 1D; *P* < 0.01). The results showed that there was no significant variation in the four indexes between the only aucubin group with the control group. As shown above, the tyloxapol can significantly increase blood lipids, while aucubin can significantly reduce high blood lipids.

#### 3.1.2 Aucubin inhibited the increase in MMP‐9, MCP‐1, apoC‐III, ICAM‐1 and VCAM‐1 induced by tyloxapol

MCP‐1 was rising when the body was reacting with inflammation. ICAM‐1 and VCAM‐1 play an important role in promoting adhesion of inflammatory sites, controlling tumour progression and metastasis and regulating immune response. The results showed that the content of MMP‐9, MCP‐1, apoC‐III, ICAM‐1 and VCAM‐1 was increasing in the group of tyloxapol while aucubin (10, 20 and 40 mg/kg) inhibited this rising trend which indicated the effects of aucubin on anti‐vascular endothelial inflammation and anti‐adhesion (Figure 1F‐J).

#### 3.1.3 Aucubin elevated the level of SOD

**Figure 2:**
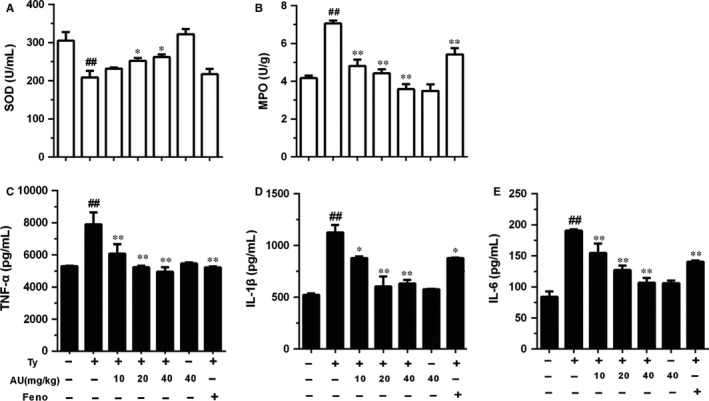
Aucubin regulated the levels of superoxide dismutase (SOD), myeloperoxidase (MPO), tumour necrosis factor receptor‐α (TNF‐α), interleukin‐1β (IL‐1β) and IL‐6 in mice. After 1 h of tyloxapol injection the serum was collected. The contents of TNF‐α, IL‐1β and IL‐6 in serum were detected by ELISA. A, The level of SOD in serum. B, The content of MPO in liver. C, The level of TNF‐α. D, The level of IL‐1β. E, The level of IL‐6. The values represent mean ± SEM of three independent experiments and differences between mean values were assessed by Student's *t* test. ^##^ *P* < 0.01 versus the control group, *P < 0.05 and **P < 0.01 versus the Ty‐treated group. The values represent mean ± SEM of three independent experiments and differences between mean values were assessed by Student's *t* test. ^##^ *P* < 0.01 versus the control group, *P < 0.05 and **P < 0.01 versus the Ty‐treated group

SOD is a free radical scavenger, which can scavenge superoxide anion free radicals in the body. Lower SOD content in the body implies that the body's ability to remove harmful free radicals is reduced. As shown in Figure 2A, SOD content decreased significantly after tyloxapol stimulation (*P* < 0.01). However, the content of SOD in aucubin groups (20 and 40 mg/kg) increased significantly (*P* < 0.01) and the application of aucubin significantly increased the SOD content, which suggested that aucubin could improve the body's antioxidant capacity.

#### 3.1.4 Aucubin reduced the increasing level of MPO induced by tyloxapol

As shown in Figure 2B, MPO content in tyloxapol group increased significantly compared to the control group (*P* < 0.01). The content of MPO in the aucubin groups (10, 20 and 40 mg/kg) and fenofibrate group significantly decreased compared with tyloxapol group (*P* < 0.01). There was no difference between the aucubin only group and the control group.

#### 3.1.5 Aucubin inhibited the increase in TNF‐α, IL‐1β and IL‐6 caused by tyloxapol

The content of pro‐inflammatory cytokines in the blood can directly reflect the immune response ability of the body. TNF‐α, IL‐1β and IL‐6 expression of mice was determined by ELISA. As shown in Figure 2C‐E, the tyloxapol group showed a significant increase in the content of TNF‐α, IL‐1β and IL‐6 (*P* < 0.01). The content of TNF‐α, IL‐1β and IL‐6 in fenofibrate group and aucubin groups (10, 20 and 40 mg/kg) significantly decreased (*P* < 0.01 or *P* < 0.05). There was no significant change in the indexes after the application of aucubin alone.

#### 3.1.6 Aucubin regulated the expression of Nrf2, HO‐1, PPARα, PPARγ in tyloxapol‐induced hyperlipaemia of mice

**Figure 3:**
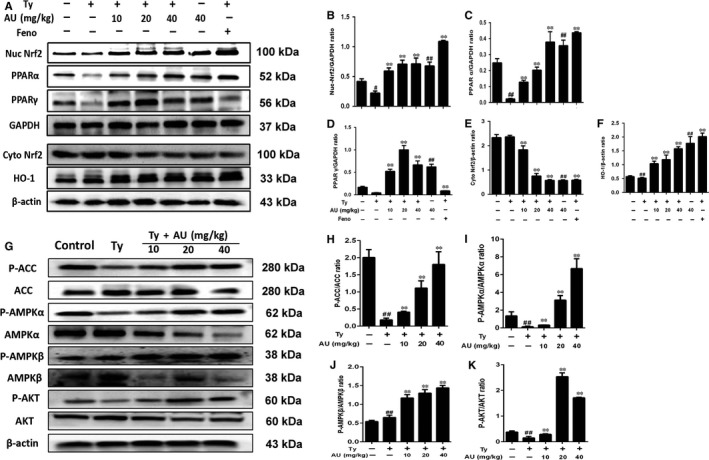
Aucubin regulated the expression of NF‐E2‐related factor 2 (Nrf2), hemeoxygenase‐1 (HO‐1), peroxisome proliferator‐activated receptor‐α (PPARα), PPARγ and the phosphorylation of acetyl‐CoA carboxylase (ACC), adenosine 5′‐monophosphate‐activated protein kinase (AMPKα), AMPKβ and protein kinase B (AKT) in tyloxapol induced hyperlipaemia of mice. C57BL/6 mice were received aucubin (10, 20, 40 mg/kg) prior to 1 h tyloxapol (500 mg/kg) injection and liver was collected for Western blot. A, The expression of Nrf2, HO‐1, PPARα and PPARγ in nucleoprotein and the expression of Nrf2, HO‐1 in cytoplasmic protein. B‐F, Relative expression levels of all proteins including Nrf2, PPARα, PPARγ in nuclear protein and Nrf2, HO‐1 in cytoplasmic protein. G, The expression of P‐ACC, P‐AMPKα, P‐AMPKβ and P‐AKT in liver of mice. H‐K, Relative expression levels of all proteins including P‐ACC, ACC, P‐AMPKα, AMPKα, P‐AMPKβ, AMPKβ, P‐AKT and AKT

As is mentioned above, when stimulated by activator, Nrf2 can transfer from cytoplasm to the nucleus and play its transcriptional activity. We examined the effect of aucubin on Nrf2 activity by Western blotting. The experimental results showed that the content of Nrf2 in the nuclear protein increased significantly and presented a dose‐dependent manner (*P* < 0.01) after the treatment of aucubin (10, 20 and 40 mg/kg). HO‐1 in the cytoplasm also increased in a dose‐independent manner (*P* < 0.01; Figure 3A, B, F). Furthermore, the results showed that aucubin also enhanced the expression of PPARα and PPARγ in the nucleus. It showed that aucubin (10, 20 and 40 mg/kg) could inhibit tyloxapol‐induced hyperlipaemia of mice via activating the Nrf2, PPARα and PPARγ into the nucleus and resisting the oxidative stress in the body (Figure 3A, C, D).

#### 3.1.7 Aucubin regulated the phosphorylation of ACC, AMPKα AMPKβ and AKT in tyloxapol‐induced hyperlipaemia of mice

As is shown in Figure 3G, H, I, J and K, aucubin could enhance the phosphorylation of ACC, AMPKα, AMPKβ and AKT significantly on tyloxapol‐induced NAFLD of mice.

#### 3.1.8 Aucubin reduced the accumulation of lipid in the liver of mice

**Figure 4:**
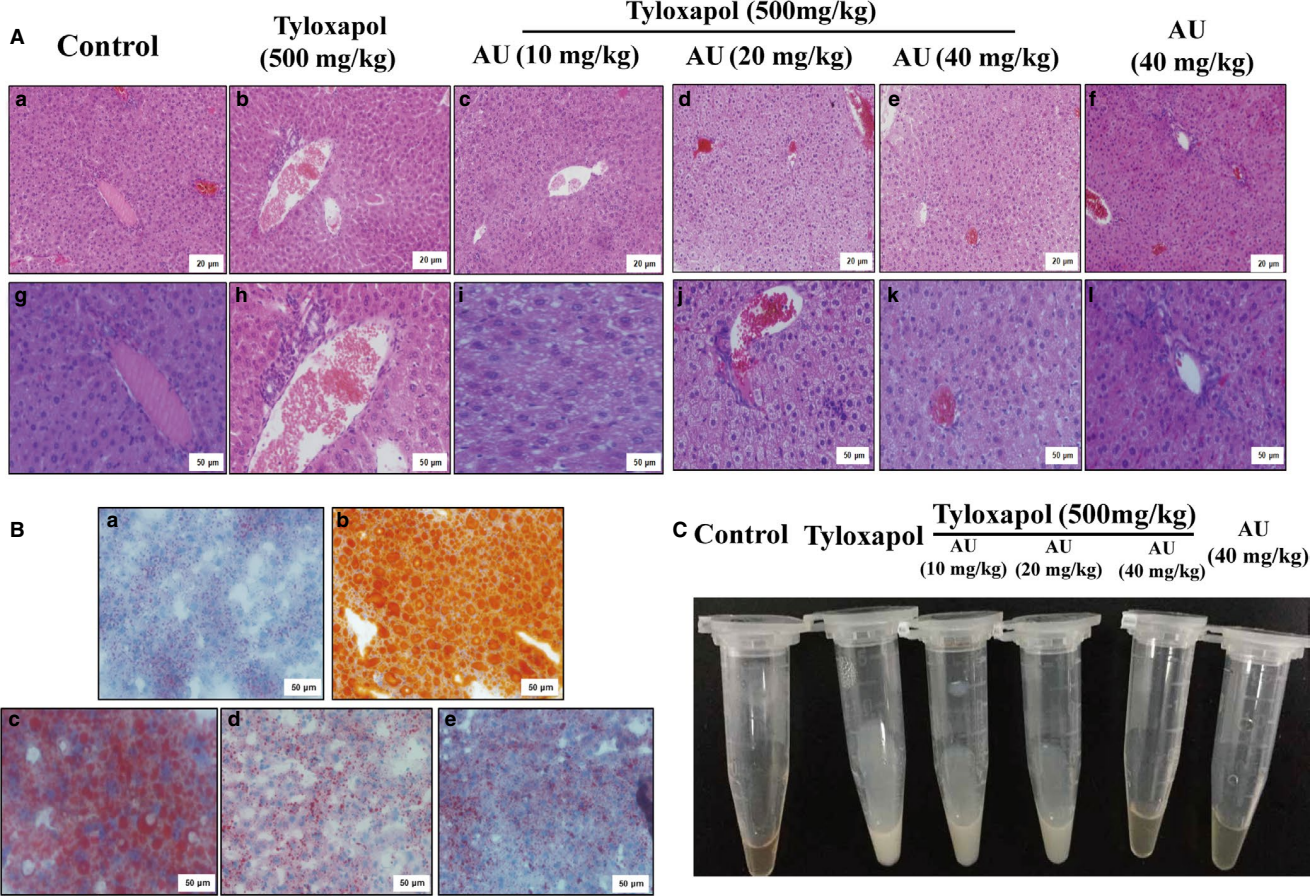
Aucubin reduced the accumulation of lipid and inflammation in liver and serum of mice. A, Haematoxylin and eosin staining of liver tissues (a, b, c, d, e, f magnification 200 × , scale bars: 20 μm; g, h, i, j, k, l magnification 400 × , scale bars: 50 μm). B, Oil Red O staining of liver tissues. a : Control group; b : Tyloxapol group; c : Ty+Aucubin (AU) (10 mg/kg); d : Ty+AU (20 mg/kg); e:Ty+AU (40 mg/kg) (magnification 200 × , scale bars: 50 μm). C, Macroscopic view of serum in different groups

Oil‐red O could dye the lipid into red, while haematoxylin dyes the cell nucleus into blue. As shown in Figure 4A, in the tyloxapol group the normal nucleus (blue) had been covered by the lipid droplets (red). The application of fenofibrate and aucubin (10, 20 and 40) can clearly reduce the fat in the liver and the nuclear structure was gradually clear.

#### 3.1.9 Aucubin reduced the inflammation damages of liver

The liver pathological injury was examined to detect the anti‐inflammation effect of aucubin. As is shown in Figure 4B(a), the liver of control group appeared clear and normal structure. Tyloxapol treatment induced the inflammation cells (such as neutrophil) infiltration and fat vacuoles gathering (Figure 4B(b)). Aucubin pre‐treatment significantly alleviate the lipid accumulation and the lobule and liver sinusoid became obvious gradually (Figure 4B(c, d and e)).

#### 3.1.10 Aucubin reduced the lipid gathering in liver and serum

As shown in Figure 4C, the serum of the control group was clear and transparent, while that of the tyloxapol group was milk fat and opaque. However, after the application of aucubin, the content of serum lipid decreased and gradually turned translucent.

As shown in Table 2, the mice weight, the liver weight and liver index of tyloxapol group all increased compared with the control group. Compared with the tyloxapol group, the liver index of three concentrations of aucubin groups (10, 20 and 40 mg/kg) decreased in different degrees which explained that aucubin could break the lipid production both in liver and blood.

**Table 2:** Liver index of each group

Group	Quantity	Weight (g)	Liver weight (g)	Liver index (%)
Control	3	18.46 ± 0.065	0.8705 ± 0.030	4.71%
Tyloxapol	3	18.42 ± 0.42	0.9561 ± 0.0301	5.2%
Ty + Aucubin (10 mg/kg)	3	19.145 ± 1.005	0.9545 ± 0.0167	5.00%
Ty + Aucubin (20 mg/kg)	3	18.385 ± 0.275	0.9041 ± 0.0854	4.91%
Ty + Aucubin (40 mg/kg)	3	19.685 ± 0.145	0.9125 ± 0.0868	4.63%
Ty + Fenofibrate	3	20.253 ± 0.137	0.9920 ± 0.0455	4.89%
Aucubin (40 mg/kg)	4	18.0775 ± 0.2375	0.8800 ± 0.002	4.87%

### 3.2 The effect of aucubin on 3T3‐L1 cell

#### 3.2.1 Aucubin affected the cell viability

**Figure 5:**
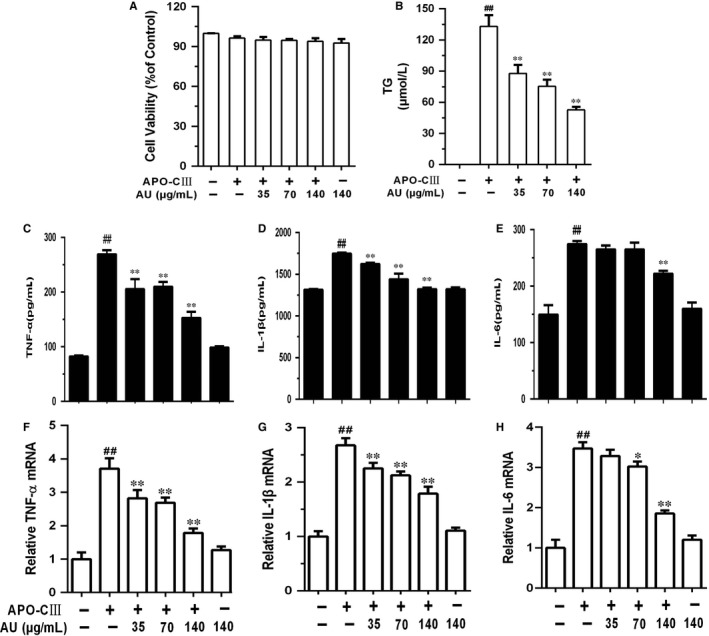
Aucubin affected on the cell viability inhibited the releasing of tumour necrosis factor receptor‐α (TNF‐α), interleukin‐1β (IL‐1β) and IL‐6 induced by apoC‐III. A, The differentiation 3T3‐L1 cells were exposed to aucubin (20, 35, 70, 140 and 280 μg/mL) in absence or presence of apoC‐III. The effect of aucubin on cell viability was detected using CCK‐8 assay. B, The differentiation 3T3‐L1 cells were exposed to aucubin (35, 70, 140 μg/mL) prior 1 h apoC‐III (100 μg/mL) stimulation. The content of triglyceride (TG) was detected by ELISA kit. C‐H, The contents and mRNA expression of TNF‐α, IL‐1β and IL‐6 were detected by ELISA and qRT‐PCR. C, The content of TNF‐α. D, The content of IL‐1β. E, The content of IL‐6. F, mRNA expression of TNF‐α. G, mRNA expression of IL‐1β. H, mRNA expression of IL‐6. The values represent mean ± SEM of three independent experiments and differences between mean values were assessed by Student's *t* test. ^##^ *P* < 0.01 versus the control group, *P < 0.05 and **P < 0.01 versus the Ty‐treated group

The differential 3T3‐L1 cells were treated with aucubin (20, 35, 70, 140 and 280 μg/mL) in the absence or presence of apoC‐III, and the effect of aucubin on cell viability was measured by CCK‐8 assay. As shown in Figure 5A, aucubin had no effect on the cell quantities and viability with or without apoC‐ III.

#### 3.2.2 Aucubin inhibited the release of TG, TNF‐α, IL‐1β and IL‐6 induced by apoC‐ III

We concluded from Figure 5A that apoC‐III could promote 3T3‐L1 cells release TG, TNF‐α, IL‐1β and IL‐6 while aucubin suppressed this increasing trend in a dose‐independent manner in Figure 5C‐H.

#### 3.2.3 Aucubin enhanced the expression of Nrf2, HO‐1, PPARα, PPARγ and P‐ACC, P‐AMPKα, P‐AMPKβ and P‐AKT in differential 3T3‐L1 cell

**Figure 6:**
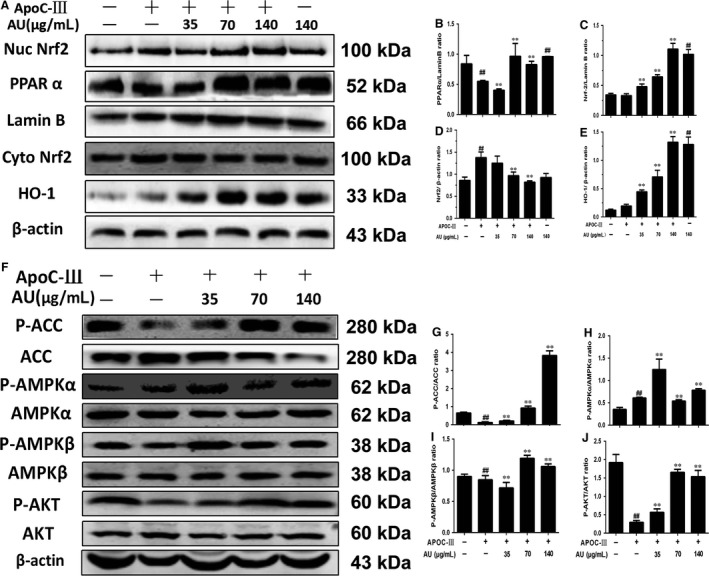
Aucubin enhanced the expression of NF‐E2‐related factor 2 (Nrf2), hemeoxygenase‐1 (HO‐1), peroxisome proliferator‐activated receptor α (PPARα), PPARγ and P‐acetyl‐CoA carboxylase (ACC), P‐adenosine 5′‐monophosphate‐activated protein kinase (AMPKα), P‐AMPKβ and P‐protein kinase B (AKT) in differential 3T3‐L1 cells. A, The expression of Nrf2, HO‐1, PPARα and PPARγ in nucleoprotein and the expression of Nrf2, HO‐1 in cytoplasmic protein. B‐E, Relative expression levels of all proteins including Nrf2, PPARα, PPARγ in nuclear protein and Nrf2, HO‐1 in cytoplasmic protein. F, The expression of P‐ACC, P‐AMPKα, P‐AMPKβ and P‐AKT in differentiation 3T3‐L1 cell in total protein. G‐J, Relative expression levels of all proteins including P‐ACC, ACC, P‐AMPKα, AMPKα, P‐AMPKβ, AMPKβ, P‐AKT and AKT. The values represent mean ± SEM of three independent experiments and differences between mean values were assessed by Student's *t* test. ^##^ *P* < 0.01 versus the control group, *P < 0.05 and **P < 0.01 versus the Ty‐treated group

In Figure 6A‐E, we could see that aucubin promoted the expression of Nrf2, HO‐1, PPARα and PPARγ, which stated that aucubin may inhibit the lipid accumulation via facilitating the transference of Nrf2 into nucleus the same as the results in vivo. Moreover, aucubin also could increase the phosphorylation level of ACC, AMPKα, AMPKβ and AKT which confirmed the results of vivo experiments (Fig. 6F‐J).

## 4 DISCUSSION

It is widely accepted and confirmed that atherosclerosis adhered to monocytes/lymphocyte activation of endothelial cells (EC) of chronic inflammatory diseases, whose early symptoms are usually hyperlipidaemia. Inflammation is a complex interaction between soluble factors and cells that can occur in any tissue to cope with trauma, infectivity, ischaemia, poisoning or autoimmune damage. Inflammation seems to be involved in all stages of atherosclerosis.^33^ When endothelial cells are activated and express chemokines, including monocyte chemoattractant protein (MCP)‐1 and interleukin (IL)‐8, as well as adhesion molecules, including ICAM‐1, VCAM‐1, nucleated cells/lymphocytes are recruited and infiltrated into the endothelium, which is involved in the formation of early fat streaks.^34^ In our study, the levels of MCP‐1, ICAM‐1 and VCAM‐1 in tyloxapol group were significantly higher than the control group while aucubin inhibited the increasing trend in a dose‐dependent manner which indicated that aucubin may control the development of hyperlipidaemia via depressing the vascular adhesion (Figure 1F, I and J). Apolipoprotein C‐III is a small surface protein found on many TRLs and is considered to be a key factor in hypertriglyceridaemia because of its inhibitory effect on apolipoprotein catabolism.^35^ ApoC‐III has been shown to be an important factor in regulating plasma triglyceride concentrations in vitro. In rodent and human subjects, increased expression of apoC‐III is associated with elevated plasma triglyceride levels, and decreased triglyceride levels.^36^, ^37^ In our study, the results showed that the level of apoC‐III was increasing with the higher TC, TG, LDL and VLDL in tyloxapol group while aucubin inhibited the rising trend (Figure 1A‐C, E and H). In vitro, lipoprotein lipase and liver lipase can be inhibited by apoC‐III via blocking the binding of apoB100 or apoE to liver receptors, delaying the clearance of very low density lipoprotein (VLDL),^38^, ^39^ so we used apoC‐III as the stimulant in differential 3T3‐L1 cells.

Some studies report pro‐inflammatory cytokines could stimulate atherosclerosis chemokines and adhesion molecules that enable early culture of monocytes and lymphocytes of endometrium. In addition, the cytokine activated macrophages and blood vessel cells of matrix metalloproteinases (MMPS) and promoted cell apoptosis. Cytokines play a potentially harmful role in advanced atherosclerosis, often causing plaques to rupture or erode more easily. The balance between pro‐inflammatory cytokines and anti‐inflammatory cytokines is a major determinant of plaque stability.^40^ Therefore, it is necessary to explore the role of inflammatory cytokines and inflammatory signalling pathways in early atherosclerosis, which would also make a contribution to understanding the mechanism of aucubin. In our study, we explored the content and mRNA expression of TNF‐α, IL‐1β and IL‐6 both in vivo and vitro. In tyloxapol induced mice and apoC‐III induced 3T3‐L1 cells, the levels of TNF‐α, IL‐1β and IL‐6 were increasing while pre‐treatment of aucubin controlled the rise (Figures 2C‐E and 5C‐H). In addition, the increasing concentration of MMP‐9 in tyloxapol‐induced mice was suppressed by aucubin in a dose‐dependent manner 9 (Figure 1F).

SOD value is an important index to measure the ability of anti‐oxidation and scavenge free radical in vivo. In our study, the SOD content in the tyloxapol group decreased significantly compared with the control group, indicating that the body's antioxidant capacity was impaired and the process of hyperlipidaemia was accelerated. However, it was found that the content of SOD increased significantly after the application of aucubin implying the gradual recovery of the body's anti‐oxidative stress ability (Figure 2A)

Transcription factor nuclear factor‐erythroid 2‐related factor 2 (Nfe2l2/Nrf2) is a basic leucine zipper transcription factor that regulates transcriptional induction of ARE‐containing genes encoding antioxidant enzymes, ubiquitin/proteasomes and chaperone and heat‐shock proteins in response to cellular stresses including ROS.^41^, ^42^

Normally, Nrf2 is localized in the cytoplasm primarily through interaction with Kelch ECH Joint Protein 1 (Keap1) and actin cytoskeleton. During exposure to electrophiles or oxidative stress, Keap1 becomes oxidized at critical cysteine residues. As a result, Nrf2 escapes Keap1 control and translocates to the nucleus, where it promotes the expression of ARE‐containing genes.^42^, ^43^, ^44^ In brief, Nrf2 protects the liver from steatosis by inhibiting lipogenesis and promoting fatty acid oxidation. This may be explained by the activation of ARE‐containing transcription factors that regulate adipocyte differentiation and adipogenesis (eg PPARγ) and by the protection against redox‐dependent inactivation of metabolic enzymes.^45^ It also has been expounded in some articles that PPAR relevant proteins play a vital role of regulating the lipid metabolism of other animals like cows.^46^, ^47^ In our study, we detected the effects of aucubin on the expression of Nrf2/HO‐1, PPARα and PPARγ both in vivo and vitro. Aucubin could significantly increase the activation of Nrf2, HO‐1, PPARα and PPARγ in mice and 3T3‐L1 cell and promote them transferring into the nucleus (Figures 3 and 6).

Besides, we also detected the effect of aucubin on the AMPK signalling pathway. Many studies have reported that AMPK could be activated by many natural products and inhibited by lipid overload, and can modulate the oxidative stress response via regulating ROS production. In our study, we found that tyloxapol and apoC‐III could obviously inhibit the phosphorylation of ACC, AMPKα and AKT while aucubin activated the phosphorylation of ACC, AMPKα, AMPKβ and AKT. As the previous study reported, oxidative stress often occurs in the progress of hyperlipaemia and even NAFLD by regulating ROS and MPO production.^48^ The level of MPO and ROS in tyloxapol‐induced mice decreased significantly compared with the control group while aucubin pre‐treatment suppressed the decreasing trend indicating that high blood lipid was accompanied by the process of oxidative stress (Figure 2B). What is more, the activity of AMPK changes with the occurrence of oxidative stress. The observation indicated that aucubin decreased the lipid accumulation and oxidative damage via regulating the expression of Nrf2, PPARα, PPARγ and AMPK relevant proteins.

Hepatic lipid level of liver can be observed through oil red O staining of frozen section. The lipid in the liver of tyloxapol group was increased significantly compared with the control group, while the lipid content decreased in different degrees after the pre‐treatment with aucubin. The liver of the aucubin alone group was basically the same as that of the control group, indicating that the application of aucubin alone could not produce toxic and adverse effects on the liver (Figure 4A).

**Figure 7:**
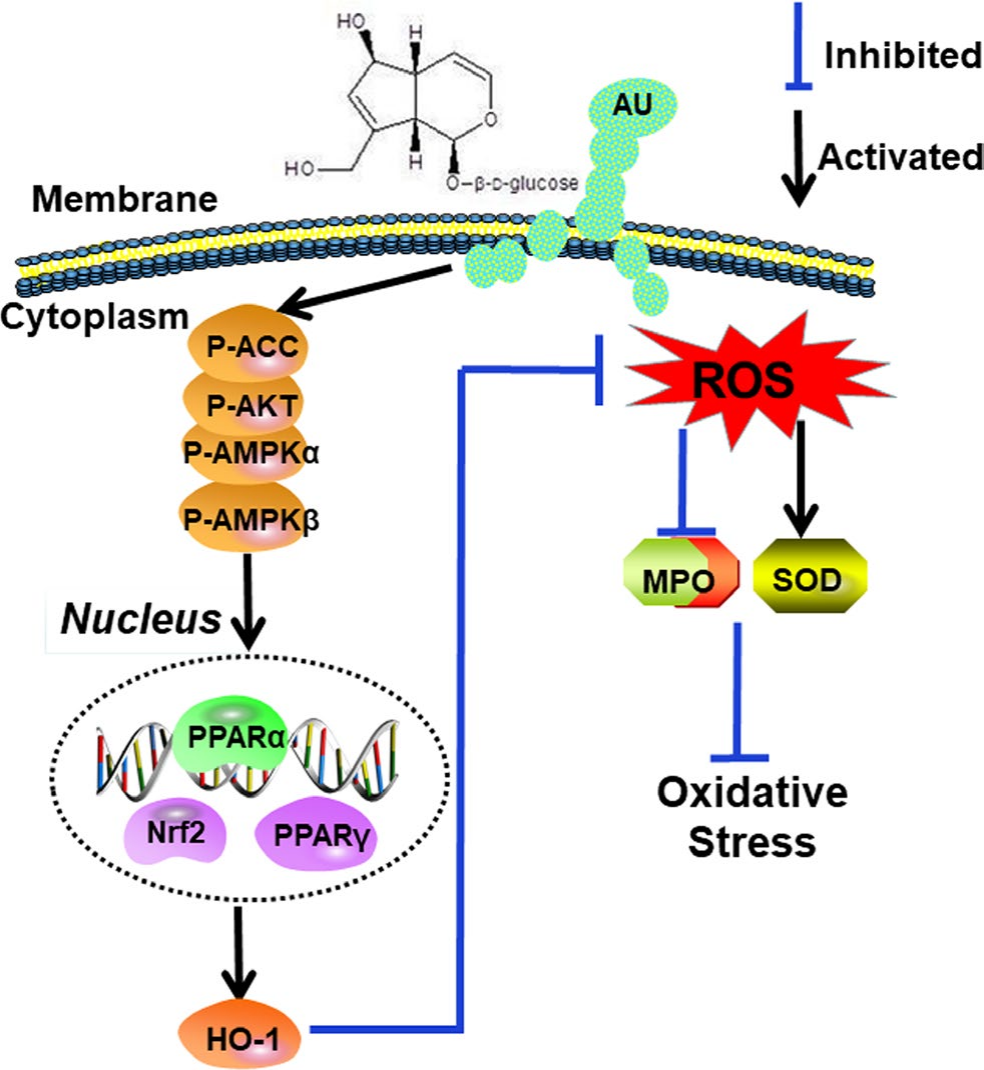
Protection effects of Aucubin (AU) against lipid accumulation and inflammation in tyloxapol induced hyperlipidaemia mice and apoC‐III induced differential 3T3‐L1 cells via activating Nrf2/hemeoxygenase‐1 (HO‐1) signalling pathway. AU aggravated the phosphorylation of acetyl‐CoA carboxylase (ACC), adenosine 5′‐monophosphate‐activated protein kinase (AMPKα), AMPKβ and protein kinase B (AKT), which contributed to up‐regulating peroxisome proliferator‐activated receptor α (PPARα) and PPARγ. On the other hand, AU alleviated the oxidative stress and inflammation response by inhibiting the release of tumour necrosis factor receptor‐α (TNF‐α), interleukin‐1β (IL‐1β), IL‐6 and ROS. Importantly, these signalling pathways play a pivotal role in suppressing lipid accumulation and oxidative stress

In conclusion, as shown in Figure 7, aucubin is capable of relieving the lipid accumulation through activating Nrf2 and PPAR both in mice and 3T3‐L1 cell. Aucubin also inhibited the release of pro‐inflammatory cytokines such as TNF‐α, IL‐1β and IL‐6 and enhanced the phosphorylation of ACC, AMPKα and AKT which were associated with oxidative stress and inflammation in hyperlipaemia. From the above, aucubin may be a potential therapeutic drug targeting at NAFLD and hyperlipaemia.

## AUTHORS’ CONTRIBUTIONS

B.Y.S., C.X.Z., Y.W. and H.H.F. conceived the study and participated in its design. B.Y.S., C.X.Z. and Z.L. performed the experimental work. B.Y.S. and Y.W. wrote the manuscript. P.Y., L.W., M.Y.J., J.Q.C. and H.H.F. participated in the writing of the manuscript and its critical review. All co‐authors revised the manuscript and approved the final submitted version.

## CONFLICT OF INTEREST

The authors declare no conflict of interest.
